# The development and validation of a prediction model for post-AKI outcomes of pediatric inpatients

**DOI:** 10.1093/ckj/sfaf007

**Published:** 2025-01-09

**Authors:** Chao Zhang, Xiaohang Liu, Ruohua Yan, Xiaolu Nie, Yaguang Peng, Nan Zhou, Xiaoxia Peng

**Affiliations:** Department of Clinical Epidemiology and Evidence-based Medicine, Beijing Children's Hospital, Capital Medical University, National Center for Children Health, Beijing, China; Department of Clinical Epidemiology and Evidence-based Medicine, Beijing Children's Hospital, Capital Medical University, National Center for Children Health, Beijing, China; Department of Clinical Epidemiology and Evidence-based Medicine, Beijing Children's Hospital, Capital Medical University, National Center for Children Health, Beijing, China; Department of Clinical Epidemiology and Evidence-based Medicine, Beijing Children's Hospital, Capital Medical University, National Center for Children Health, Beijing, China; Department of Clinical Epidemiology and Evidence-based Medicine, Beijing Children's Hospital, Capital Medical University, National Center for Children Health, Beijing, China; Department of Nephrology, Beijing Children's Hospital, Capital Medical University, National Center for Children Health, Beijing, China; Department of Clinical Epidemiology and Evidence-based Medicine, Beijing Children's Hospital, Capital Medical University, National Center for Children Health, Beijing, China

**Keywords:** acute kidney injury, dialysis, hospital mortality, pediatric, prediction model

## Abstract

**Background:**

Acute kidney injury (AKI) is common in hospitalized children. A post-AKI outcomes prediction model is important for the early detection of important clinical outcomes associated with AKI so that early management of pediatric AKI patients can be initiated.

**Methods:**

Three retrospective cohorts were set up based on two pediatric hospitals in China, in which 8205 children suffered AKI during hospitalization. Two clinical outcomes were evaluated, i.e. hospital mortality and dialysis within 28 days after AKI occurrence. A Genetic Algorithm was used for feature selection, and a Random Forest model was built to predict clinical outcomes. Subsequently, a temporal validation set and an external validation set were used to evaluate the performance of the prediction model. Finally, the stratification ability of the prediction model for the risk of mortality was compared with a commonly used mortality risk score, the pediatric critical illness score (PCIS).

**Results:**

The prediction model performed well for the prediction of hospital mortality with an area under the receiver operating curve (AUROC) of 0.854 [95% confidence interval (CI) 0.816–0.888], and the AUROC was >0.850 for both temporal and external validation. For the prediction of dialysis, the AUROC was 0.889 (95% CI 0.871–0.906). In addition, the AUROC of the prediction model for hospital mortality was superior to that of PCIS (*P *< .0001 in both temporal and external validation).

**Conclusions:**

The new proposed post-AKI outcomes prediction model shows potential applicability in clinical settings.

KEY LEARNING POINTS **What was known:**

- Prediction models to early detect important clinical events for adult inpatients who developed AKI have been reported.
- Nevertheless, there have been few studies on such prediction models for pediatric inpatients.

**This study adds:**

- We established and validated a post-AKI outcomes prediction model for pediatric inpatients based on routinely collected data using machine learning method.
- The model performed well in predicting hospital mortality and dialysis.

**Potential impact:**

- The new proposed model showed the potential applicability to assist the early management of pediatric AKI patients and optimize delivery of healthcare services and therefore improve the outcomes.

## INTRODUCTION

Acute kidney injury (AKI) is an adverse event characterized by a rapid decrease in kidney function. Various studies have reported data on its incidence and mortality. AKI occurs in approximately 1%–26.9% of hospitalized children [1–3]. In China, the overall incidence of AKI is about 20% in hospitalized children [4]. It has been reported that AKI is associated with poor prognosis in pediatric patients, including the length of hospital stay, chronic kidney disease and mortality [5–8]. The Assessment of Worldwide Acute Kidney Injury, Renal Angina, and Epidemiology (AWARE) study, an observational study of 4683 children, reported a higher 28-day mortality in patients with severe AKI than in those without (11% vs 2.5%) [1].

Due to the poor outcomes associated with AKI, several prediction models of post-AKI outcomes have been reported, which can detect post-AKI outcomes in adult patients [9–11]. To date, the majority of post-AKI outcomes prediction studies in the pediatric population have focused on biomarkers [12, 13]. However, these biomarkers are not routinely examined. To our knowledge, there are no prediction models for post-AKI mortality or dialysis of children based on routinely collected data. Considering the children's characteristics during a growth and development stage, there is an urgent need for the development of prediction models that help to stratify the risk of prognostics of pediatric inpatients suffering from AKI. In this way, the model could assist in the early management of pediatric AKI patients and optimize the delivery of healthcare services, and therefore improve the outcomes [9].

In recent years, machine learning methods have been widely developed and applied in diverse medical fields [14]. Due to their ability to capture nonlinearities and complex interactions among multiple features, machine learning methods have advantages in dealing with “big data” [15]. Previous studies have shown that machine learning models such as Random Forest and extreme gradient boosting (XGBoost) can outperform traditional statistical methods in predicting the development and outcomes of AKI [9, 16–18].

The primary goal in the present study is to develop a prediction model of post-AKI outcomes using machine learning algorithms for pediatric inpatients, which is expected to output a risk of in-hospital mortality or dialysis upon AKI.

## MATERIALS AND METHODS

### Study design and participants

The present study was performed in accordance with the recommendations laid out in the World Medical Association Declaration of Helsinki. Ethics approval was obtained from the Institutional Review Board (IRB) of the Beijing Children's Hospital [IRB No. (2022)-E-207-Y]. Written consent was waived by the IRB because only retrospective data was used.

This study was performed using three retrospective cohorts from two pediatric hospitals in China. Firstly, the derivation cohort consisted of all patients hospitalized at Beijing Children's Hospital, Capital Medical University from 2015–21, with which we trained and performed an internal validation of the prediction model. Then, the temporal validation cohort consisted of all patients hospitalized at Beijing Children's Hospital, Capital Medical University from 2022–23, with which we performed a validation of the prediction model. Lastly, the external validation cohort consisted of all patients admitted to intensive care units of the Children's Hospital of Zhejiang University School of Medicine from 2010–19, with which we performed another validation. Both Beijing Children's Hospital and Children's Hospital of Zhejiang University School of Medicine are tertiary care university teaching hospitals, located in North China and East China, respectively.

The inclusion criteria were defined as following: (i) aged 28 days or more; (ii) pediatric inpatients with at least two creatinine tests; (iii) pediatric inpatients diagnosed as AKI during hospitalization. The exclusion criteria included: (i) developed AKI within 24 h after admission; (ii) diagnosed as end-stage renal disease at admission. The same selection criteria of participants were followed in two hospitals, covering three cohorts in the present study.

This manuscript is written following the Transparent Reporting of a Multivariable Prediction Model for Individual Prognosis or Diagnosis guideline (TRIPOD) checklist [19].

### Data extraction and preprocess

All variables were extracted from electronic medical records (EMR), including demographics, comorbidities at admission and laboratory testing data. Comorbidities were determined by International Classification of Diseases, 10th Revision, Clinical Modification codes. As for repeated measurements of laboratory tests, we only considered the most recent measurement values before AKI occurrence [20]. Missing continuous variables were imputed by median value [21]. Variables with missing rate higher than 20% were excluded. The amount of missing data is presented in Supplementary data, Table S1.

### Selection of features

In order to reduce the number of available features, the key features were selected based on Genetic Algorithm (GA) [22, 23]. GA is a machine learning technique in the domain of evolutionary optimization inspired by biological evolutionary processes [24, 25]. At the beginning, we randomly generate binary strings for *N* individuals to build up the initial population; the lengths of the strings are equal to the number of total features. Each string represents a feature subset and the values at each position in the string are coded as either presence or absence of a particular feature [26]. The preferable feature subsets are the individuals that have higher fitness values. Then, new subsets are generated through crossover and mutation operation. Mutation changes some of the values (thus adding or deleting features) in a subset randomly. Crossover combines different features from a pair of subsets into a new subset. The algorithm is an iterative process in which each successive generation is produced by the members of the current generation.

### The definition of AKI

AKI status was labeled according to Kidney Disease: Improving Global Outcomes (KDIGO) criteria [27]. Due to the relative sparsity of urine output data on the general hospital wards, urine output criteria for AKI were not considered. According to KDIGO guidelines, an increase in serum creatinine of 0.3 mg/dL (26.5 μmol/L) within 48 h or an increase in serum creatinine of 1.5× the baseline creatinine level within 7 days was identified as AKI [28, 29]. Baseline creatinine was determined by the first creatinine measurement upon admission.

### Outcomes

Two clinical outcomes were evaluated: (i) hospital mortality within 28 days after AKI occurrence; or (ii) dialysis within 28 days after AKI occurrence, including hemodialysis, peritoneal dialysis, hemodiafiltration and continuous renal replacement therapy. In-hospital death and dialysis were extracted from EMR and confirmed by a senior nephrologist (N.Z.). In addition, only outcomes that occurred before discharge were considered.

### Model training and validating

A Random Forest algorithm was employed to predict the outcomes of AKI inpatients. Hyper-parameters of the Random Forest model were fine-tuned with grid search method and cross-validation. Platt scaling was used to recalibrate the model [30].

To make the full use of available data, we did not split the derivation set. Instead, we trained model with the entire derivation set and used bootstrap method for internal validation [31]. Additionally, a temporal validation set and an external validation set were used to evaluate the performance of the prediction model. Sensitivity (also known as recall), specificity, positive predictive value (also known as precision), negative predictive value, the area under the receiver-operating curve (AUROC), the area under the precision–recall curve (AUPR), calibration intercept and calibration slope were calculated to assess the model performance [32, 33]. The cut-off values were chosen for the model to achieve a precision of 0.5 in the training set. To obtain stable estimations of performance indices, the performance indices and confidence intervals (CI) were calculated using bootstrap method with 200 replications.

To further demonstrate the stratification ability of the prediction model for mortality risk, we also included a commonly used risk score for comparison. Currently, there are three widely used scoring criteria to assess mortality risk for pediatric patients, such as pediatric risk of mortality (PRISM) [34], pediatric index of mortality (PIM) [35] and pediatric critical illness score (PCIS) [36]. PCIS score was finally chosen for comparison, given that the necessary information to calculate PRISM and PIM was not available in the dataset. We performed DeLong's test for the statistical comparison of AUROCs between the machine learning model and PCIS. In addition, the Kaplan–Meier method was used to construct survival curves. The output risks of the prediction model were categorized as low risk when being below the cut-off value. Otherwise, the outputs were considered high risk. Additionally, a PCIS score <80 was classified as high risk, while score ≥80 was deemed low risk.

### Model interpretability

The Random Forest model cannot directly offer any explanations regarding the clinical meaning of features. In this way, we utilized the SHapley Additive exPlanation (SHAP) value, derived from game theory, to determine the importance of features in our prediction model [37]. It was calculated by taking the average marginal contribution of all possible coalitions for a feature value [38, 39]. At the same time, the local SHAP plots were figured to show the individual-level SHAP value and the importance of each variable in the prediction model.

### Statistical analysis

Data preprocessing was conducted using SAS (version 9.4). Machine learning models were built and assessed using Python (version 3.10.4). SAS (version 9.4) and R (version 4.1.0) were used for statistical analyses. Specifically, categorical variables were summarized in terms of counts and percentages (%), whereas continuous variables were described using median values and interquartile ranges (IQR).

## RESULTS

### The baseline characteristics of participants

**Figure 1:**
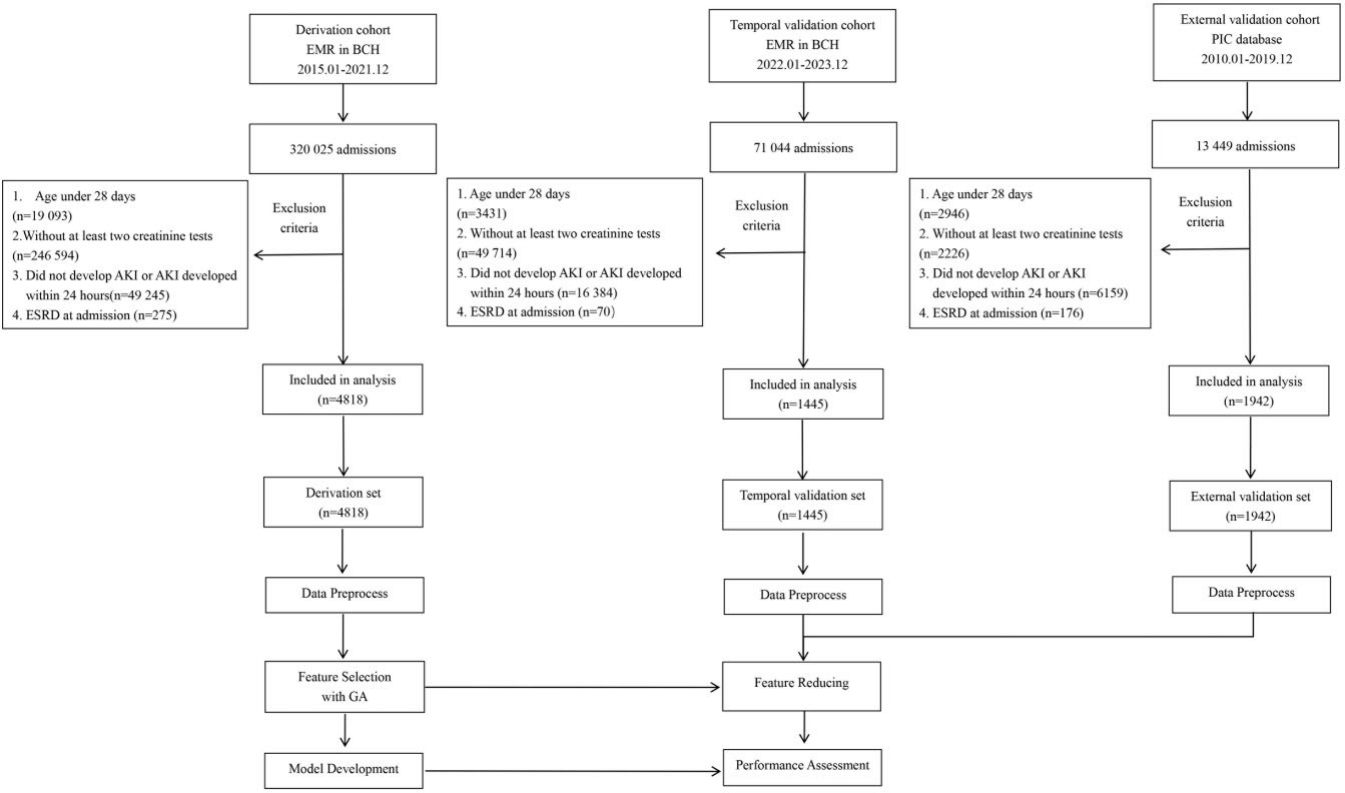
Flow chart of the study design. BCH: Beijing Children's Hospital, Capital Medical University; ESRD: end-stage renal disease; PIC: Pediatric Intensive Care.

According to the selection criteria of participants, 4818 of 320 025 children diagnosed as AKI were selected based on the derivation cohort. In addition, 1445 children occurred AKI were filtered from 71 044 inpatients based on the temporal validation cohort. At the same time, 1942 cases of AKI were filtered from 13 449 inpatients of the external validation cohort and were used to assess the prediction model. The flow diagram for participants is shown in Fig. 1.

As shown in Table 1, 58.2% of 4818 AKI inpatients in the derivation cohort were boys, the median PCIS score was 86 (IQR 82–90) and the median age was 4.5 (IQR 1.7–8.8) years. The case numbers (proportion) at stage 1, stage 2 and stage 3 of AKI were 3407 (70.7%), 903 (18.7%) and 508 (10.5%), respectively. In addition, 37.3% and 6.3% of children were exposed to non-steroidal anti-inflammatory drugs or angiotensin-converting enzyme inhibitors, respectively. There does not seem to be much difference between the baseline characteristics in the derivation cohort and temporal cohort, but the baseline characteristics in the derivation cohort seems different from those in the external validation cohort. In the external validation cohort, the pediatric inpatients had a lower median age of 1.6 (IQR 0.6–4.2) years. The laboratory values in the external validation cohort reflect that these pediatric patients were more critical cases because they all stayed in intensive care units. They had a higher median creatinine of 45.0 (IQR 38.0–58.0) μmol/L, a higher median lactic dehydrogenase of 410 (IQR 297–580) U/L and a higher median creatinine kinase of 201 (IQR 72–613) U/L. There were also differences between the spectrum of comorbidities and usage of drugs in the patients of the derivation cohort and those of the external validation cohort.

**Table 1:** The demographic and clinical characteristics of participants.

	Derivation cohort	Temporal validation cohort	External validation cohort
Number of hospitalizations	4818	1445	1942
Gender (male), *N* (%)	2803 (58.2)	818 (56.6)	1017 (52.4)
Age (years), median (IQR)	4.5 (1.7, 8.8)	6.4 (3.1, 10.5)	1.6 (0.6, 4.2)
PCIS, median (IQR)	86 (82, 90)	86 (82, 90)	84 (82, 88)
AKI stage, *N* (%)			
Stage 1	3407 (70.7)	1121 (77.6)	1280 (65.9)
Stage 2	903 (18.7)	218 (15.1)	452 (23.3)
Stage 3	508 (10.5)	106 (7.3)	210 (10.8)
Comorbidities, *N* (%)			
Diabetes	173(3.6)	45 (3.1)	0 (0)
Heart failure	71 (1.5)	53 (3.7)	6 (0.3)
Hypertension	196 (4.1)	119 (8.2)	0 (0)
Liver disease	41 (0.9)	22 (1.5)	0 (0)
Rheumatic	86 (1.8)	48 (3.3)	0(0)
Sepsis	278 (5.8)	133 (9.2)	7 (0.4)
Drugs, *N* (%)			
ACEI	303 (6.3)	132 (9.1)	56 (2.9)
Acyclovir	498 (10.3)	169 (11.7)	56 (2.9)
AGs	179 (3.7)	70 (4.8)	3 (0.2)
Anti-tumor	1504 (31.2)	474 (32.8)	33 (1.7)
ARB	23 (0.5)	9 (0.6)	1 (0.1)
Diuretics	288 (6.0)	134 (9.3)	251 (12.9)
NSAIDs	1798 (37.3)	592 (41.0)	296 (15.2)
Laboratory data, median (IQR)			
Creatinine (μmol/L)	33.9 (24.3, 51.2)	36.5 (25.2, 58.3)	45.0 (38.0, 58.0)
eGFR (mL/min/1.73 m^2^)	96.1 (61.4, 128.8)	101.5 (65.8, 135.9)	52.3 (27.8, 70.3)
White blood cell count (10^9^/L)	7.3 (3.8, 11.6)	6.9 (3.4, 11.3)	10.8 (7.7, 14.7)
Phosphorus (mmol/L)	1.5 (1.2, 1.7)	1.5 (1.2, 1.8)	1.5 (1.2, 1.7)
Platelet (10^9^/L)	284 (203, 369)	287 (205, 367)	234 (153, 332)
Albumin (g/L)	35.5 (30.9, 39.5)	35.9 (31.6, 39.7)	38.8 (34.6, 42.2)
Lactic dehydrogenase (U/L)	297 (226, 477)	278 (215, 415)	410 (297, 580)
Total protein (g/dL)	59.7 (52.7, 66.3)	60.3 (53.9, 66.5)	58.8 (53.3, 65.1)
Cholesterol (mmol/L)	3.7 (2.9, 4.7)	3.7 (3.0, 4.8)	3.0 (2.3, 3.9)
Creatine kinase (U/L)	42 (21, 111)	34 (19, 85)	201 (72, 613)
Creatine kinase isoenzyme (U/L)	18 (12, 28)	16 (12, 25)	36 (23, 59)
Vital signs, median (IQR)			
Diastolic pressure (mmHg)	100 (90, 110)	99 (92, 108)	100 (90, 109)
Heart rate (bpm)	120 (100, 139)	118 (97, 135)	126 (108, 140)
Systolic pressure (mmHg)	60 (52, 70)	60 (52, 68)	57 (48, 65)
Temperature (°C)	37.2 (36.8, 38.0)	37.0 (36.7, 38.0)	36.8 (36.5, 37.2)

ACEI: angiotensin-converting enzyme inhibitor; AGs: amino glycosides; ARB: angiotensin receptor blocker; eGFR: estimated glomerular filtration rate; NSAIDs: non-steroidal anti-inflammatory drugs.

### Outcomes

As shown in Table 2, mortality within 28 days after AKI occurrence was 3.4% and 3.6% in the derivation cohort and temporal validation cohort, respectively. In addition, the external validation cohort had a higher hospital mortality (6.8%) because the children admitted to intensive care unit usually had more severe conditions.

**Table 2:** Outcome incidence in different cohorts, *n* (%).

	Derivation cohort	Temporal validation cohort	External validation cohort
Number of hospitalizations	4818	1445	1942
Death, *N* (%)	165 (3.4)	52 (3.6)	130 (6.8)
Dialysis, *N* (%)	434 (9.0)	149 (10.3)	5 (0.3)

As for dialysis within 28 days, 9.0% and 10.3% of AKI patients underwent dialysis in the derivation cohort and temporal validation cohort, respectively. Unfortunately, the external validation set did not provide dialysis operation information. We have attempted to detect the outcome of dialysis by analyzing the prescription data, only five pediatric AKI patients who performed dialysis were detected.

### Prediction of mortality within 28 days after AKI occurrence

Thirty-two features (shown in Supplementary data, Table S1) were selected for the prediction of mortality by the GA.

**Figure 2:**
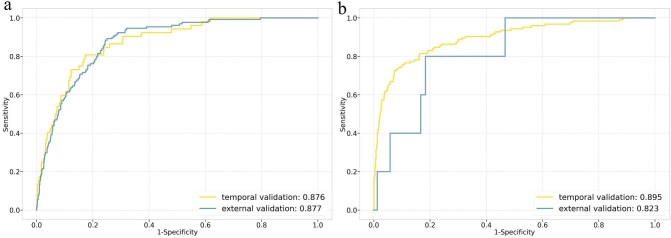
Receiver operating curve of the machine learning model. (**a**) Death. (**b**) Dialysis.

The receiver operating curve of the prediction model is shown in Fig. 2a (model developed with all data in the derivation cohort). Furthermore, we performed a bootstrap method to assess performance indices in three cohorts. The model performed robustly in all cohorts, with: AUROC = 0.854 (0.816, 0.888) in the derivation cohort; AUROC = 0.875 (0.859, 0.891) in the temporal validation cohort; and AUROC = 0.858 (0.833, 0.878) in the external validation cohort. Details of the performance metrics can be found in Table 3.

**Table 3:** The performance metrics of the prediction model.

	Internal validation		Temporal validation		External validation	
Performance metrics	Hospital mortality	Dialysis	Hospital mortality	Dialysis	Hospital mortality	Dialysis
AUROC (95% CI)	0.854 (0.816, 0.888)	0.889 (0.871, 0.906)	0.875 (0.859, 0.891)	0.879 (0.874, 0.885)	0.858 (0.833, 0.878)	0.782 (0.730, 0.828)
AUPR (95% CI)	0.256 (0.178, 0.332)	0.580 (0.525, 0.630)	0.271 (0.228, 0.310)	0.595 (0.576, 0.614)	0.314 (0.286, 0.340)	0.009 (0.006, 0.017)
Precision (PPV) (95% CI)	0.131 (0.104, 0.160)	0.232 (0.199, 0.267)	0.153 (0.137, 0.170)	0.246 (0.232, 0.260)	0.168 (0.144, 0.197)	0.005 (0.004, 0.006)
Sensitivity (95% CI)	0.730 (0.631, 0.822)	0.876 (0.831, 0.912)	0.738 (0.673, 0.808)	0.850 (0.826, 0.872)	0.868 (0.807, 0.931)	0.930 (0.800, 1.000)
Specificity (95% CI)	0.828 (0.801, 0.854)	0.711 (0.681, 0.742)	0.847 (0.823, 0.868)	0.700 (0.673, 0.725)	0.688 (0.619, 0.750)	0.482 (0.407, 0.549)
NPV (95% CI)	0.989 (0.984, 0.993)	0.983 (0.977, 0.988)	0.989 (0.986, 0.992)	0.976 (0.973, 0.979)	0.987 (0.980, 0.992)	1.000 (NA)
Calibration slope (95% CI)	0.908 (0.557, 1.226)	1.234 (1.060, 1.441)	1.021 (0.861, 1.171)	1.153 (1.079, 1.231)	1.208 (1.067, 1.401)	0.765 (0.550, 0.955)
Calibration intercept (95% CI)	–0.062 (–0.409, 0.309)	0.004 (–0.219, 0.229)	0.021 (–0.125, 0.178)	0.109 (0.029, 0.191)	0.101 (–0.076, 0.319)	–4.208 (–4.329, –4.089)

Confidence intervals were constructed using bootstrap method.

NPV: negative predictive value; PPV: positive predictive value.

**Figure 3:**
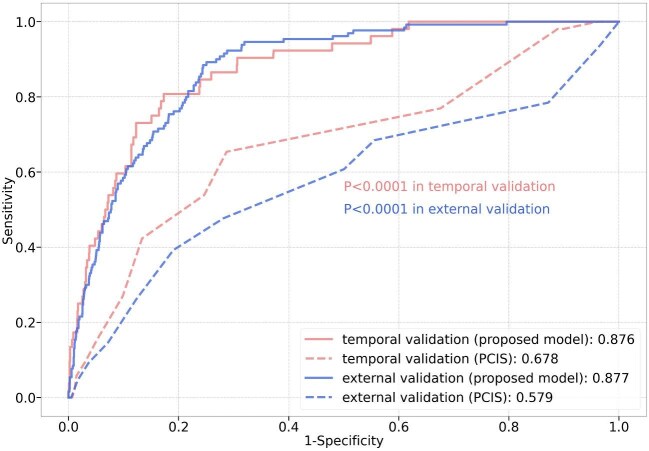
Receiver operating curve of the machine learning model and PCIS score. DeLong's test was used for the statistical comparison.

**Figure 4:**
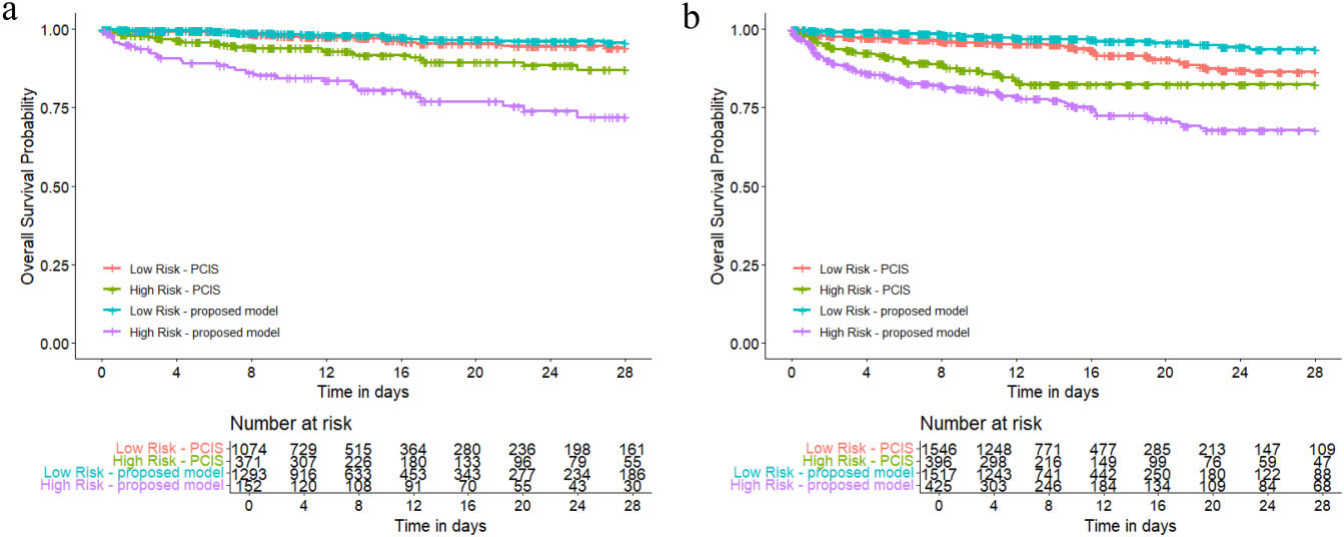
Survival curve of the machine learning model and PCIS score. (**a**) Survival curve of children in the temporal validation cohort. (**b**) Survival curve of children in the external validation cohort. The low- and high-risk patients were classified by the prediction model or PCIS score.

The receiver operating curves of both the machine learning model and PCIS are shown in Fig. 3. In both temporal validation cohort and external validation cohort, the new proposed model outperformed PCIS score (0.876 vs 0.678 in temporal validation; 0.877 vs 0.579 in external validation). The DeLong's test shows that the AUROC of the machine learning model is statistically significantly higher than that of the PCIS score in both temporal validation and external validation (*P *< .0001). The survival curves are drawn in Fig. 4 for a more intuitive comparison. The survival curves of inpatients were divided into two groups by PCIS scores in red (low risk, PCIS ≥ 80) and green (high risk, PCIS < 80). The survival curves of inpatients were also grouped according to the risks generated by the machine learning model in blue (low risk) and purple (high risk). From the survival curves, it can be observed that the prediction model did better on mortality risk stratification, compared with PCIS in both cohorts.

To more closely approximate a probability, the output generated by the model was recalibrated via Platt scaling. Supplementary data, Fig. S1 shows the calibration plots of the model and Table 3 shows the calibration slope and intercept calculated by bootstrap method.

**Figure 5:**
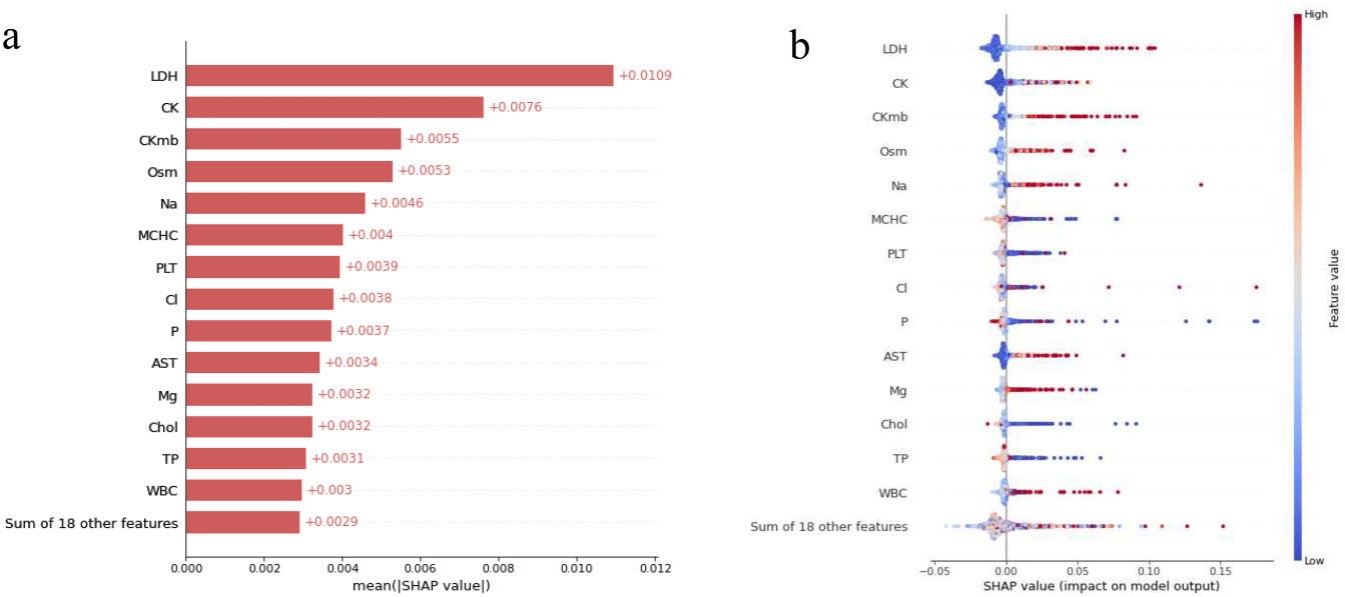
Model explanation on mortality prediction by the SHAP method. (**a**) SHAP summary bar plot. The bar plot shows the importance of each feature based on the absolute mean value of the SHAP value. (**b**) SHAP beeswarm plot. In the beeswarm plot, each sample is represented as a single dot, and the position is determined by its SHAP value, while the color indicates its original feature value. Red signifies a higher feature value and blue signifies a lower feature value. LDH: lactic dehydrogenase; CK: creatinine kinase; CKmb: creatinine kinase MB isoenzyme; OSM: osmolality; Na: sodium; MCHC: mean corpuscular hemoglobin concentration; PLT: platelet; Cl: chloride; P: phosphorus; AST: aspartate transaminase; Mg: magnesium; Chol: cholesterol; TP: total protein; WBC: white blood cell.

Then, we used SHAP method to find the features that were important for the prediction of mortality. In Fig. 5a, the strongest predictors of hospital mortality are listed in the SHAP summary bar plot, including lactic dehydrogenase, creatine kinase, creatine kinase MB isoenzyme, osmolality, sodium, mean corpuscular hemoglobin concentration, platelet, chloride, phosphorus and aspartate aminotransferase. The SHAP beeswarm summary plot (Fig. 5b) demonstrates the influence of each variable in terms of its impact on model predictions. On average, higher lactic dehydrogenase, creatine kinase, creatine kinase MB isoenzyme, osmolality, sodium, chloride and aspartate aminotransferase are associated with a greater risk of hospital mortality. Instead, lower mean corpuscular hemoglobin concentration and platelet are associated with a greater risk of hospital mortality.

In addition, we quantified the feature's contribution to each individual prediction by analyzing the local SHAP values (Supplementary data, Fig. S2). Supplementary data, Fig. S2a shows the individual local SHAP values of a child who died 14 days after AKI development. The cholesterol, lactic dehydrogenase, total protein, aspartate aminotransferase, sodium and creatinine kinase MB isoenzyme pushed the prediction towards the higher risk of mortality. Supplementary data, Fig. S2b shows the individual local SHAP values of another child. This child suffered abnormal chloride, sodium, osmolality, magnesium and lactic dehydrogenase, and died 1 day after developed AKI.

### Prediction of dialysis

Supplementary data, Table S1 lists the feature selection results of the GA. There were 27 features selected for the prediction of dialysis.

The receiver operating curve of the prediction model to predict dialysis is shown in Fig. 2b. Furthermore, the results of bootstrap estimation can be found in Table 3. The model performed robustly in all cohorts, with: AUROC = 0.889 (0.871, 0.906) in the derivation cohort; AUROC = 0.879 (0.874, 0.885) in the temporal validation cohort; and AUROC = 0.782 (0.730, 0.828) in the external validation cohort. The AUPR and precision in external validation cohort is extremely low because only five inpatients who went on dialysis were extracted from the Pediatric Intensive Care database.

**Figure 6:**
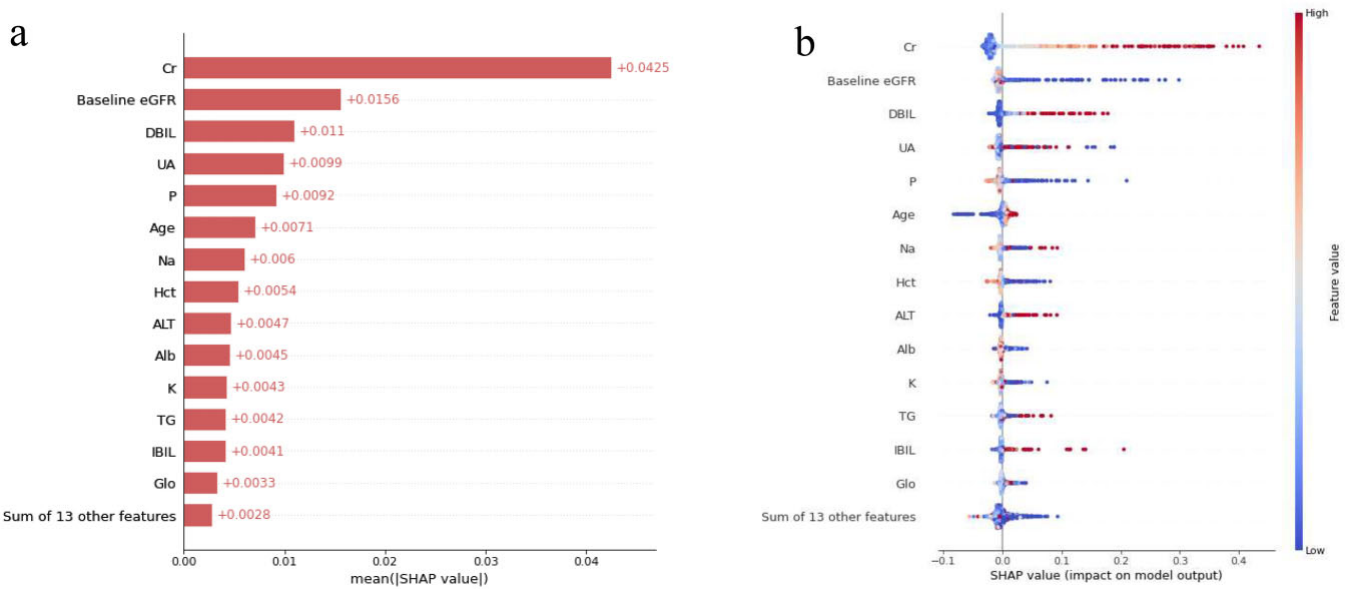
Model explanation on dialysis prediction by the SHAP method. (**a**) SHAP summary bar plot. The bar plot shows the importance of each feature based on the absolute mean value of the SHAP value. (**b**) SHAP beeswarm plot. In the beeswarm plot, each sample is represented as a single dot, and the position is determined by its SHAP value, while the color indicates its original feature value. Red signifies a higher feature value and blue signifies a lower feature value. Cr: creatinine; eGFR: estimated glomerular filtration rate; DBIL: direct bilirubin; UA: urea acid; P: phosphorus; Na: sodium; Hct: hematocrit; ALT: alanine aminotransferase; Alb: albumin; K: potassium; TG: triglyceride; IBIL: indirect bilirubin; Glo: globulin content.

In Fig. 6a, the strongest predictors of dialysis are listed in the SHAP summary bar plot, including creatinine, baseline eGFR, direct bilirubin, urea acid, phosphorus, age, sodium, hematocrit, alanine aminotransferase and albumin. The SHAP beeswarm summary plot (Fig. 6b) demonstrates the influence of each variable in terms of its impact on model predictions. On average, higher creatinine, direct bilirubin, urea acid, age, sodium and alanine aminotransferase are associated with a greater risk of dialysis. Lower baseline eGFR, phosphorus, hematocrit and albumin are associated with a greater risk of dialysis. In addition, we quantified the feature's contribution to each individual prediction by analyzing the local SHAP values (Supplementary data, Fig. S3).

## DISCUSSION

In the present study, a prediction model of post-AKI outcomes for hospitalized children was developed based on EMR data. Although there are studies have developed post-AKI outcomes prediction models for adult inpatients, studies focus on pediatric population are still limited. To our best knowledge, this is the first prediction model to predict post-AKI hospital mortality and dialysis for pediatric inpatients.

The first strength of the study is that the prediction model was developed for all hospitalized children based one a large-scale cohort. And the data used in this study were routinely collected health data, facilitating its generalization in the future.

The second strength is that the GA algorithm was used to select import features, although stepwise regression and tree-based (Boruta, Random Forest, etc.) algorithms were more popular methods for feature selection in previous clinical prediction models. Nevertheless, both have obvious flaws. The stepwise regression approach is limited by the fact that it commonly trapped in local optima [40], and the tree-based algorithms have the bias tending to select continuous variables and therefore have risk of neglecting the categorical ones [41, 42]. Several methodology studies have suggested that the GA algorithm is superior to stepwise regression and tree-based methods in feature selection tasks.

The third strength of the present study is that the developed prediction model shows robustness both in derivation cohort and validation cohorts. Despite the variability in baseline characteristics—e.g. age, the spectrum of comorbidities at admission and drug exposures between different cohorts, the prediction obtained AUROC of 0.875 and 0.858 in temporal validation cohort and external validation cohort on hospital mortality prediction, respectively. It also obtained AUROC of 0.879 and 0.782 in temporal validation cohort and external validation cohort on dialysis prediction, respectively. The performance metrics closely align with those observed in the internal validation, supporting the model's robustness and potential applicability across diverse clinical settings.

It is widely acknowledged that the mechanisms underlying predictions made by machine learning models are complex and challenging to comprehend, which impedes the integration of such models into clinical practice. Consequently, the fourth strength of this study is our endeavor to elucidate the clinical implications of prediction models. As shown in Fig. 5, the SHAP values demonstrate that the developed prediction model covered a range of well-established risk factors, such as lactic dehydrogenase, creatinine kinase, creatinine kinase MB isoenzyme, osmolality, sodium, etc., all of which exhibit higher global average absolute SHAP values than other features. Some features should be noticed that too high values or low values are both risk factors, including phosphorus and magnesium. This finding is consistent with some previous studies [43, 44]. In addition, low cholesterol is found to be related to hospital mortality in AKI inpatients, which has not attracted widespread attention. The association between cholesterol and hospital mortality needs further research to determine whether it is a real important risk factor or a confounder [45–47]. Overall, it is essential to evaluate the risk factors collectively and to understand the nonlinear relation between risk features and outcomes.

There are some limitations associated with this study. First, the model trained with retrospective cohort data necessitates further validated in the prospective studies. Second, only serum creatinine was used to define AKI due to data unavailability, by which AKI incidence could be potentially underestimated compared with the AKI diagnoses that include urine criteria. Third, the external validation set provided incomplete dialysis operation information and only five pediatric AKI patients who performed dialysis were detected, which is too low to support a reasonable evaluation. Fourth, there are still more than 20 features in the prediction model, although we performed feature selection through the GA algorithm, which may be a barrier to use of the model in clinical practice. Fortunately, the important predictors in our model are routinely collected.

## CONCLUSION

In summary, this study introduced a machine learning approach for the prediction of post-AKI outcomes for hospitalized children. Validated with both temporal and external datasets, this model successfully identified children at risk of hospital mortality or dialysis, demonstrating its potential applicability in clinical settings.

## Supplementary Material

sfaf007_Supplemental_Files

## Data Availability

The data underlying this article will be shared on reasonable request to the corresponding author.

## Notes

The authors declare that they have no relevant financial interests.
