# Supplementary material for: The development and validation of a prediction model for post-AKI outcomes of pediatric inpatients
Source: Clin Kidney J. 2025 Jan 9;18(2):sfaf007. doi: 10.1093/ckj/sfaf007 (PMC11843026; doi:10.1093/ckj/sfaf007)
Supplement: sfaf007_Supplemental_Files [file sfaf007_supplemental_files.zip › 859 Supplement-revised.docx]

**The development and validation of a prediction model for post-AKI outcomes of pediatric inpatients**

Chao Zhang^1^, Xiaohang Liu^1^, Ruohua Yan^1^, Xiaolu Nie^1^, Yaguang Peng^1^, Nan Zhou^2^, Xiaoxia Peng^1^

1 Department of Clinical Epidemiology and Evidence-based Medicine, Beijing Children’s Hospital, Capital Medical University, National Center for Children Health, Beijing, China

2 Department of Nephrology, Beijing Children’s Hospital, Capital Medical University, National Center for Children Health, Beijing, China

**Supplementary Materials**

| Figure S1. Calibration curve | **2** |
| --- | --- |
| Figure S2. Local explanation on mortality prediction by the SHAP method. | **3** |
| Figure S3. Local explanation on dialysis prediction by the SHAP method. | **4** |
| Table S1. The list of variables. | **5** |
| Table S2. The performance of prediction model in subgroups. | **8** |





**Figure S1:** **Calibration curve in derivation cohort.**

The predictions of in-hospital death and dialysis were categorized into 3 groups due to the small number of positive samples. Within each group, the mean of the risk predictions corresponded to the percentage of positive labels in that group. The diagonal line indicates ideal calibration.


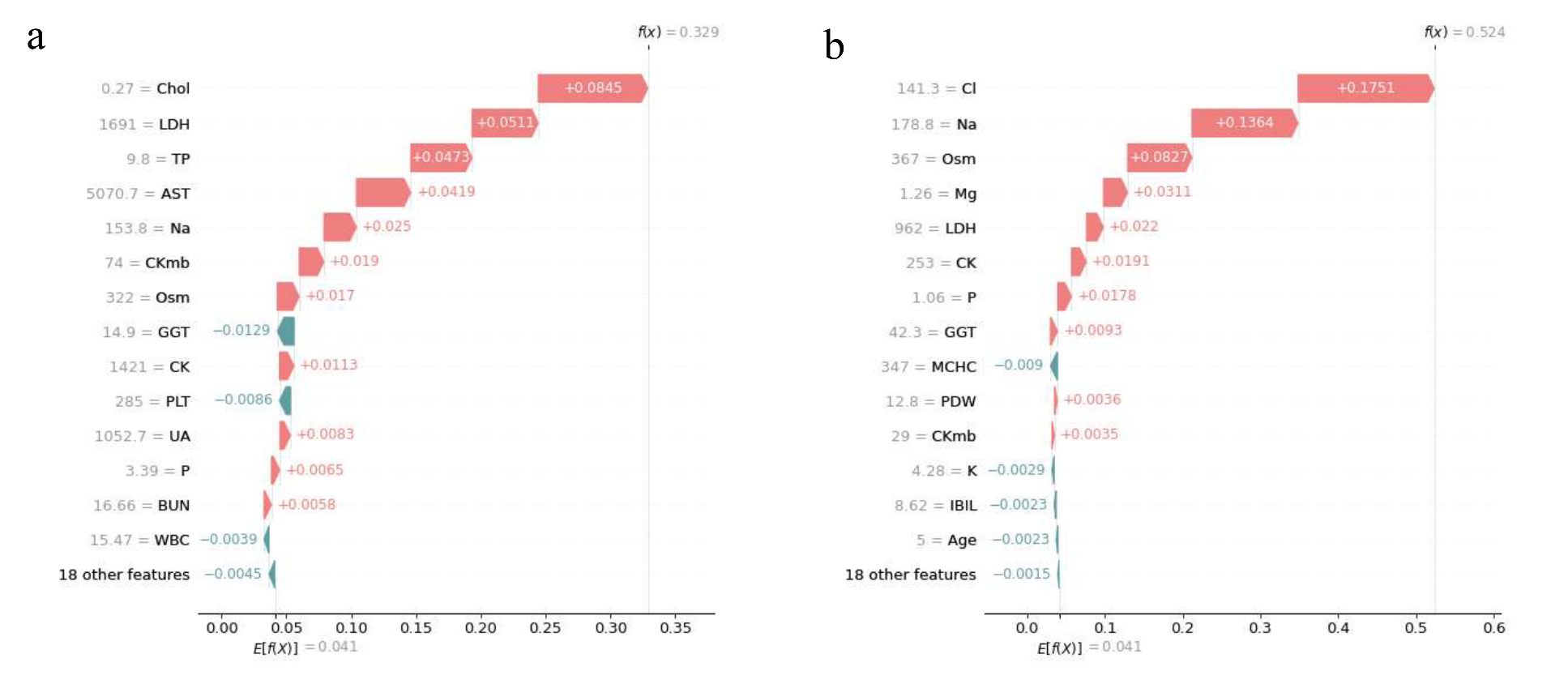


**Figure S2:** **Local explanation on mortality prediction by the SHAP method.**

(a) a child dead 14 days after developing AKI. (b) a child dead 1 day after developing AKI.

AST: aspartate aminotransferase; BUN: blood urea nitrogen; Chol: cholesterol; CK: creatine kinase; CKmb: creatine kinase MB isoenzyme; Cl: chloride; TP: total protein; GGT: gamma-glutamyl transferase; IBIL: indirect bilirubin; LDH: l[actic dehydrogenase](https://www.baidu.com/s?wd=lactic%20dehydrogenase%E7%BF%BB%E8%AF%91&rsv_idx=2&tn=baiduhome_pg&usm=1&ie=utf-8&rsv_pq=c40cceff001ae08a&oq=%E4%B9%B3%E9%85%B8%E8%84%B1%E6%B0%A2%E9%85%B6%E8%8B%B1%E6%96%87&rsv_t=d793NzThIW9eB9vfs5LnWpu6xzGvj5V0vvGEiHH7rRZvGXk9lIPHlicD5m%2Ft%2BvSvfiuB&sa=re_fy_huisou); MCHC: mean corpuscular hemoglobin concentration; Mg: magnesium; Na: sodium; Neut: neutrophil count; Osm: osmolality; P: phosphorus; PDW: platelet distribution width; PLT: platelet; UA: urea acid; WBC: white blood cell.


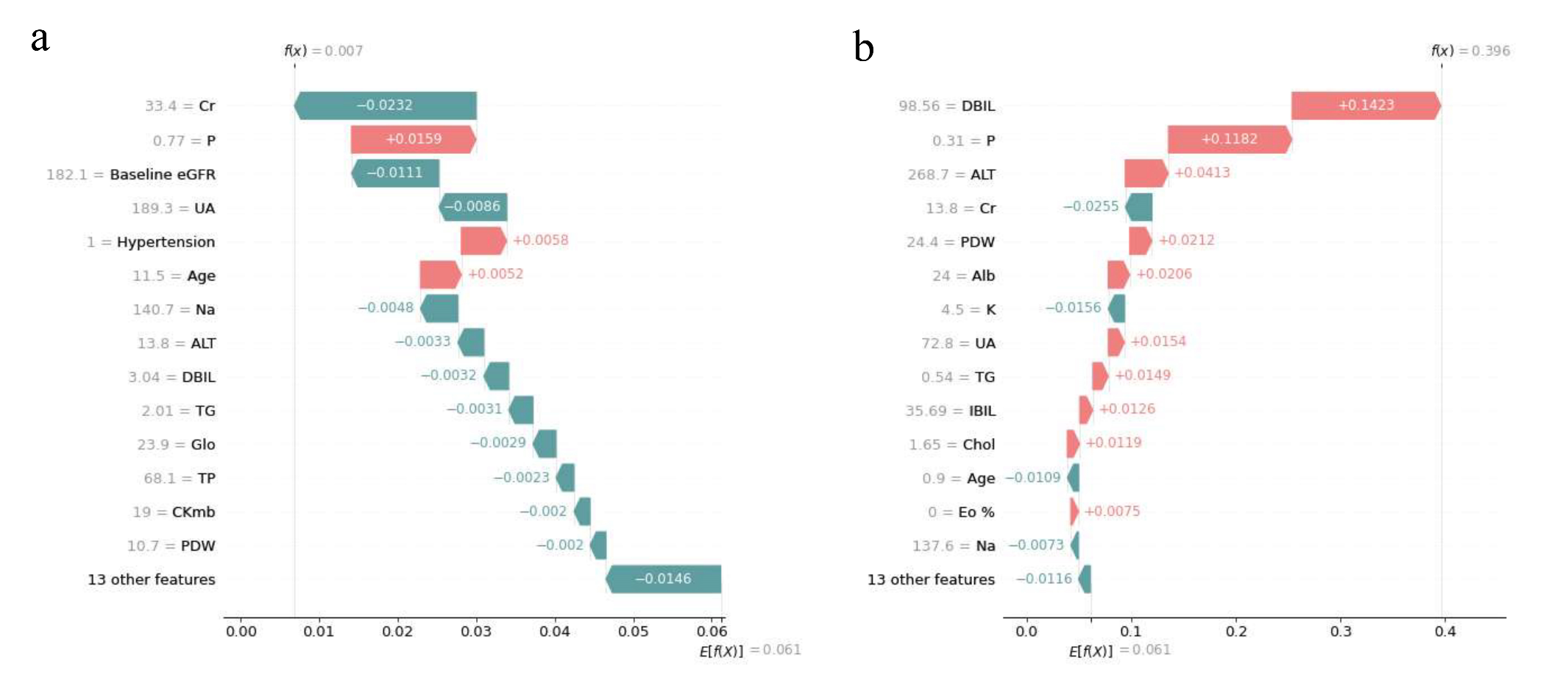


**Figure S3:** **Local explanation on dialysis prediction by the SHAP method.**

(a) a child did not go on dialysis. (b) a child performed dialysis.

Alb: albumin; ALT: alanine aminotransferase; Chol: cholesterol; CKmb: creatine kinase MB isoenzyme; Cr: creatinine; DBIL: direct bilirubin; eGFR: estimated glomerular filtration rate; EO: eosinophil; Glo: globulin content; IBIL: indirect bilirubin; K: potassium; Na: sodium; P: phosphorus; PDW: platelet distribution width; TG: triglyceride; TP: total protein; UA: urea acid.

**Table S1. The list of variables**

| **Type** | **Features** | **Features selected for death outcome** | **Features selected for dialysis**  **outcome** | **Derivation**  **cohort**  **missing data** | **Temporal validation cohort**  **missing data** | | **External validation cohort**  **missing data** |
| --- | --- | --- | --- | --- | --- | --- | --- |
| **Demographics** | Age | √ | √ | 0(0.0%) | 0(0.0%) | | 0(0.0%) |
|  | Gender | √ |  | 0(0.0%) | 0(0.0%) | | 0(0.0%) |
| **Acute kidney injury**  **Laboratory values** | AKI stage defined by KDIGO | √ |  | 0(0.0%) | 0(0.0%) | | 0(0.0%) |
|  | Albumin |  | √ | 99(2.05%) | 25(1.73%) | | 13(0.67%) |
|  | Anion gap |  |  | 3(0.06%) | 1(0.07%) | | 336(17.3%) |
|  | Alanine aminotransferase |  | √ | 73(1.52%) | 21(1.45%) | | 1(0.05%) |
|  | Alkaline phosphatase |  |  | 92(1.91%) | 25(1.73%) | | 13(0.67%) |
|  | Aspartate aminotransferase | √ |  | 71(1.47%) | 21(1.45%) | | 13(0.67%) |
|  | Baseline estimated glomerular filtration rate |  | √ | 0(0.0%) | 0(0.0%) | | 0(0.0%) |
|  | Basophil count | √ | √ | 301(6.25%) | 41(2.84%) | | 22(1.13%) |
|  | Basophil percentage | √ | √ | 301(6.25%) | 41(2.84%) | | 4(0.21%) |
|  | Blood urea nitrogen | √ |  | 0(0.0%) | 0(0.0%) | | 0(0.0%) |
|  | Calcium | √ |  | 5(0.1%) | 1(0.07%) | | 21(1.08%) |
|  | Chloride | √ |  | 1(0.02%) | 0(0.0%) | | 25(1.29%) |
|  | Creatine kinase | √ |  | 97(2.01%) | 27(1.87%) | | 22(1.13%) |
|  | Creatine kinase MB isoenzyme | √ | √ | 325(6.75%) | 89(6.16%) | | 22(1.13%) |
|  | Creatinine |  | √ | 0(0.0%) | 0(0.0%) | | 0(0.0%) |
|  | Cholesterol | √ | √ | 123(2.55%) | 43(2.98%) | | 21(1.08%) |
|  | Cholinesterase |  |  | 366(7.6%) | 102(7.06%) | | 13(0.67%) |
|  | Direct bilirubin |  | √ | 106(2.2%) | 25(1.73%) | | 14(0.72%) |
|  | Eosinophil count |  | √ | 301(6.25%) | 41(2.84%) | | 22(1.13%) |
|  | Eosinophil percentage |  | √ | 301(6.25%) | 41(2.84%) | | 4(0.21%) |
|  | Estimated glomerular filtration rate |  |  | 0(0.0%) | 0(0.0%) | | 0(0.0%) |
|  | Gamma-glutamyl transferase | √ |  | 100(2.08%) | 25(1.73%) | | 13(0.67%) |
|  | Globulin content |  | √ | 101(2.1%) | | 28(1.94%) | 13(0.67%) |
|  | Hematocrit |  | √ | 304(6.31%) | | 41(2.84%) | 1(0.05%) |
|  | Hemoglobin | √ |  | 296(6.14%) | | 41(2.84%) | 26(1.34%) |
|  | Indirect bilirubin | √ | √ | 757(15.71%) | | 25(1.73%) | 20(1.03%) |
|  | L[actic dehydrogenase](https://www.baidu.com/s?wd=lactic%20dehydrogenase%E7%BF%BB%E8%AF%91&rsv_idx=2&tn=baiduhome_pg&usm=1&ie=utf-8&rsv_pq=c40cceff001ae08a&oq=%E4%B9%B3%E9%85%B8%E8%84%B1%E6%B0%A2%E9%85%B6%E8%8B%B1%E6%96%87&rsv_t=d793NzThIW9eB9vfs5LnWpu6xzGvj5V0vvGEiHH7rRZvGXk9lIPHlicD5m%2Ft%2BvSvfiuB&sa=re_fy_huisou) | √ |  | 97(2.01%) | | 26(1.8%) | 22(1.13%) |
|  | Magnesium | √ |  | 106(2.2%) | | 27(1.87%) | 198(10.2%) |
|  | Mean corpuscular volume |  |  | 296(6.14%) | | 41(2.84%) | 1(0.05%) |
|  | Mean corpuscular hemoglobin |  | √ | 296(6.14%) | | 41(2.84%) | 1(0.05%) |
|  | Mean corpuscular hemoglobin  concentration | √ |  | 296(6.14%) | | 41(2.84%) | 1(0.05%) |
|  | Mean platelet volume |  |  | 326(6.77%) | | 41(2.84%) | 19(0.98%) |
|  | Osmolality | √ |  | 5(0.1%) | | 1(0.07%) | 1642(84.55%) |
|  | Platelet | √ | √ | 296(6.14%) | | 41(2.84%) | 1(0.05%) |
|  | Platelet distribution width | √ | √ | 340(7.06%) | | 41(2.84%) | 18(0.93%) |
|  | Phosphorus | √ | √ | 94(1.95%) | | 19(1.31%) | 21(1.08%) |
|  | Potassium | √ | √ | 3(0.06%) | | 1(0.07%) | 25(1.29%) |
|  | Red blood cell |  |  | 296(6.14%) | | 41(2.84%) | 1(0.05%) |
|  | Red blood cell distribution width | √ | √ | 297(6.16%) | | 41(2.84%) | 1(0.05%) |
|  | Sodium | √ | √ | 3(0.06%) | | 1(0.07%) | 25(1.29%) |
|  | Total bilirubin |  |  | 106(2.2%) | | 25(1.73%) | 26(1.34%) |
|  | Total bile acid |  |  | 100(2.08%) | | 25(1.73%) | 21(1.08%) |
|  | Total protein | √ | √ | 756(15.69%) | | 27(1.87%) | 13(0.67%) |
|  | Triglyceride |  | √ | 159(3.3%) | | 59(4.08%) | 21(1.08%) |
|  | Urea acid | √ | √ | 100(2.08%) | | 27(1.87%) | 13(0.67%) |
|  | White blood cell | √ |  | 296(6.14%) | | 41(2.84%) | 1(0.05%) |
| **Comorbidities** | Diabetes |  |  | 0(0.0%) | | 0(0.0%) | 0(0.0%) |
|  | Heart failure | √ |  | 0(0.0%) | | 0(0.0%) | 0(0.0%) |
|  | Hypertension | √ | √ | 0(0.0%) | | 0(0.0%) | 0(0.0%) |
|  | Liver disease | √ | √ | 0(0.0%) | | 0(0.0%) | 0(0.0%) |
|  | Rheumatic | √ |  | 0(0.0%) | | 0(0.0%) | 0(0.0%) |
|  | Sepsis |  |  | 0(0.0%) | | 0(0.0%) | 0(0.0%) |

**Table S2. The performance of prediction model in subgroups.**

| **Age <= 2 years** | | | | | | |
| --- | --- | --- | --- | --- | --- | --- |
| **Outcome** | **AUROC**  **[95% CI]** | **AUPR**  **[95% CI]** | **Precision (PPV)**  **[95% CI]** | **Sensitivity**  **[95% CI]** | **Specificity**  **[95% CI]** | **NPV**  **[95% CI]** |
| Hospital  Mortality | 0.800  [0.741, 0.852] | 0.218  [0.131, 0.317] | 0.128  [0.086, 0.180] | 0.703  [0.571, 0.852] | 0.748  [0.693, 0.796] | 0.980  [0.969, 0.991] |
| Dialysis | 0.872  [0.818, 0.921] | 0.501  [0.386, 0.623] | 0.140  [0.105, 0.177] | 0.865  [0.760, 0.964] | 0.632  [0.574, 0.690] | 0.986  [0.976, 0.996] |
| **Age > 2 years** | | | | | | |
| **Outcome** | **AUROC**  **[95% CI]** | **AUPR**  **[95% CI]** | **Precision (PPV)**  **[95% CI]** | **Sensitivity**  **[95% CI]** | **Specificity**  **[95% CI]** | **NPV**  **[95% CI]** |
| Hospital  Mortality | 0.871  [0.824, 0.918] | 0.295  [0.181, 0.396] | 0.138  [0.101, 0.171] | 0.731  [0.615, 0.834] | 0.868  [0.845, 0.889] | 0.991  [0.987, 0.995] |
| Dialysis | 0.893  [0.872, 0.913] | 0.603  [0.535, 0.664] | 0.229  [0.201, 0.262] | 0.900  [0.856, 0.937] | 0.661  [0.625, 0.697] | 0.983  [0.977, 0.989] |
| **Male** | | | | | | |
| **Outcome** | **AUROC**  **[95% CI]** | **AUPR**  **[95% CI]** | **Precision (PPV)**  **[95% CI]** | **Sensitivity**  **[95% CI]** | **Specificity**  **[95% CI]** | **NPV**  **[95% CI]** |
| Hospital  Mortality | 0.877  [0.832, 0.917] | 0.298  [0.206, 0.400] | 0.144  [0.110, 0.183] | 0.770  [0.645, 0.889] | 0.831  [0.803, 0.858] | 0.990  [0.983, 0.995] |
| Dialysis | 0.902  [0.878, 0.923] | 0.579  [0.502, 0.646] | 0.200  [0.170, 0.236] | 0.916  [0.870, 0.958] | 0.649  [0.615, 0.691] | 0.988  [0.981, 0.994] |
| **Female** | | | | | | |
| **Outcome** | **AUROC**  **[95% CI]** | **AUPR**  **[95% CI]** | **Precision (PPV)**  **[95% CI]** | **Sensitivity**  **[95% CI]** | **Specificity**  **[95% CI]** | **NPV**  **[95% CI]** |
| Hospital  Mortality | 0.815  [0.762, 0.870] | 0.202  [0.123, 0.287] | 0.118  [0.082, 0.162] | 0.643  [0.520, 0.800] | 0.838  [0.810, 0.870] | 0.986  [0.980, 0.993] |
| Dialysis | 0.872  [0.839, 0.905] | 0.587  [0.507, 0.667] | 0.209  [0.169, 0.244] | 0.863  [0.806, 0.909] | 0.657  [0.618, 0.697] | 0.979  [0.969, 0.986] |
| **AKI stage 1** | | | | | | |
| **Outcome** | **AUROC**  **[95% CI]** | **AUPR**  **[95% CI]** | **Precision (PPV)**  **[95% CI]** | **Sensitivity**  **[95% CI]** | **Specificity**  **[95% CI]** | **NPV**  **[95% CI]** |
| Hospital  Mortality | 0.831  [0.781, 0.886] | 0.194  [0.113, 0.277] | 0.108  [0.077, 0.144] | 0.674  [0.552, 0.821] | 0.849  [0.823, 0.873] | 0.990  [0.985, 0.995] |
| Dialysis | 0.878  [0.849, 0.904] | 0.542  [0.447, 0.627] | 0.178  [0.150, 0.213] | 0.847  [0.788, 0.898] | 0.705  [0.673, 0.744] | 0.984  [0.977, 0.989] |
| **AKI stage 2&3** | | | | | | |
| **Outcome** | **AUROC**  **[95% CI]** | **AUPR**  **[95% CI]** | **Precision (PPV)**  **[95% CI]** | **Sensitivity**  **[95% CI]** | **Specificity**  **[95% CI]** | **NPV**  **[95% CI]** |
| Hospital  Mortality | 0.869  [0.827, 0.910] | 0.344  [0.228, 0.460] | 0.178  [0.132, 0.224] | 0.772  [0.655, 0.880] | 0.798  [0.760, 0.834] | 0.984  [0.976, 0.992] |
| Dialysis | 0.888  [0.858, 0.916] | 0.634  [0.550, 0.704] | 0.241  [0.207, 0.283] | 0.949  [0.907, 0.984] | 0.514  [0.472, 0.561] | 0.984  [0.971, 0.995] |

Confidence intervals were constructed using bootstrap method.

AUROC: area under receiver-operating curve; AUPR: area under precision-recall curve; CI: confidence interval; NPV: negative predictive value; PPV: positive predictive value.
